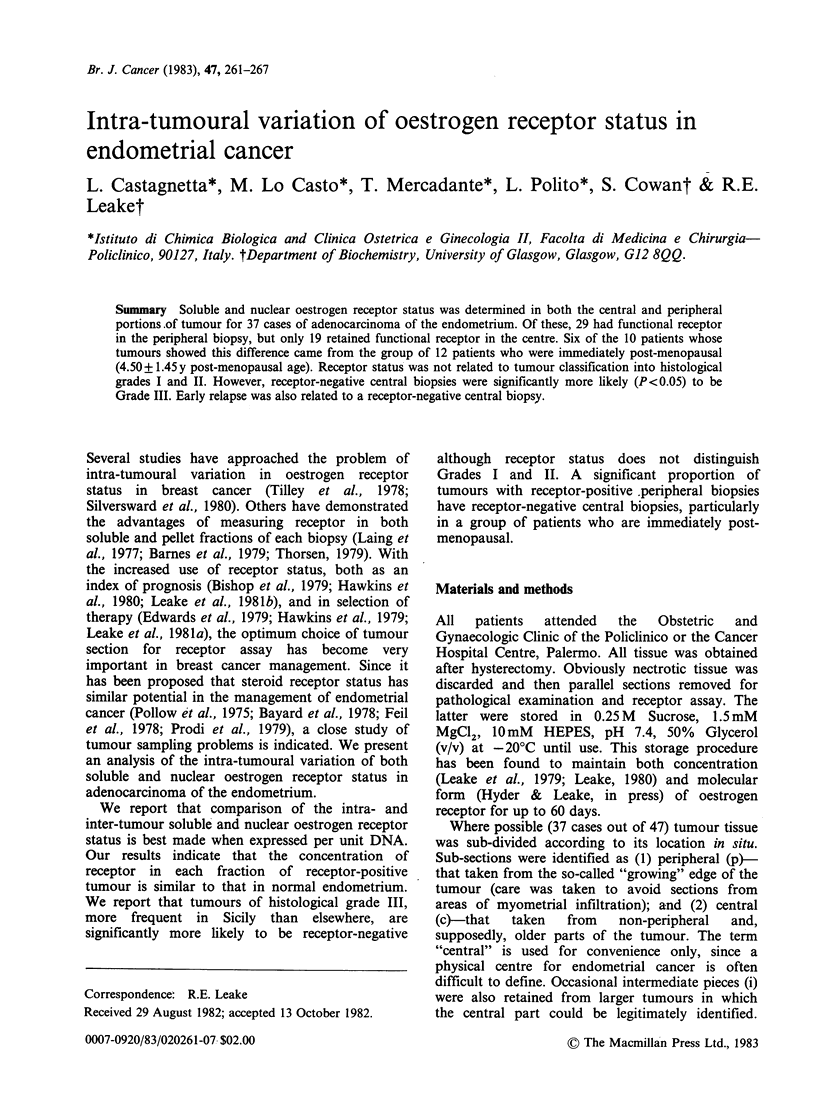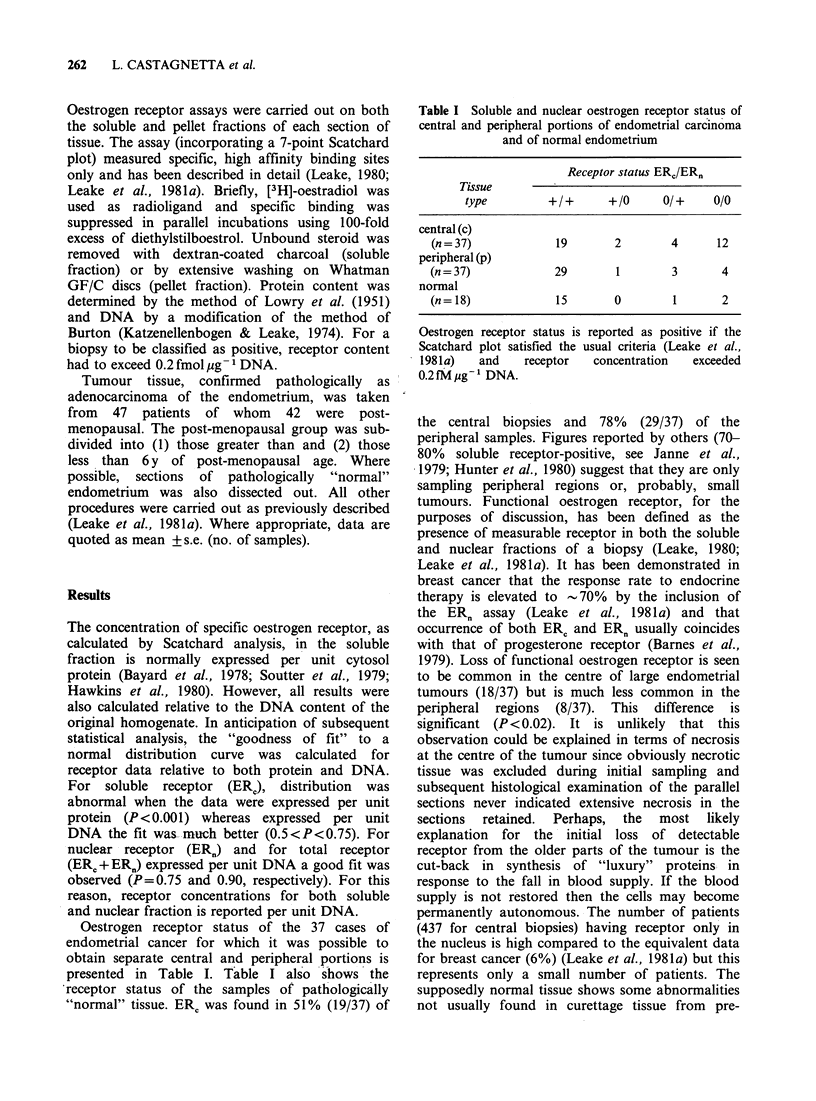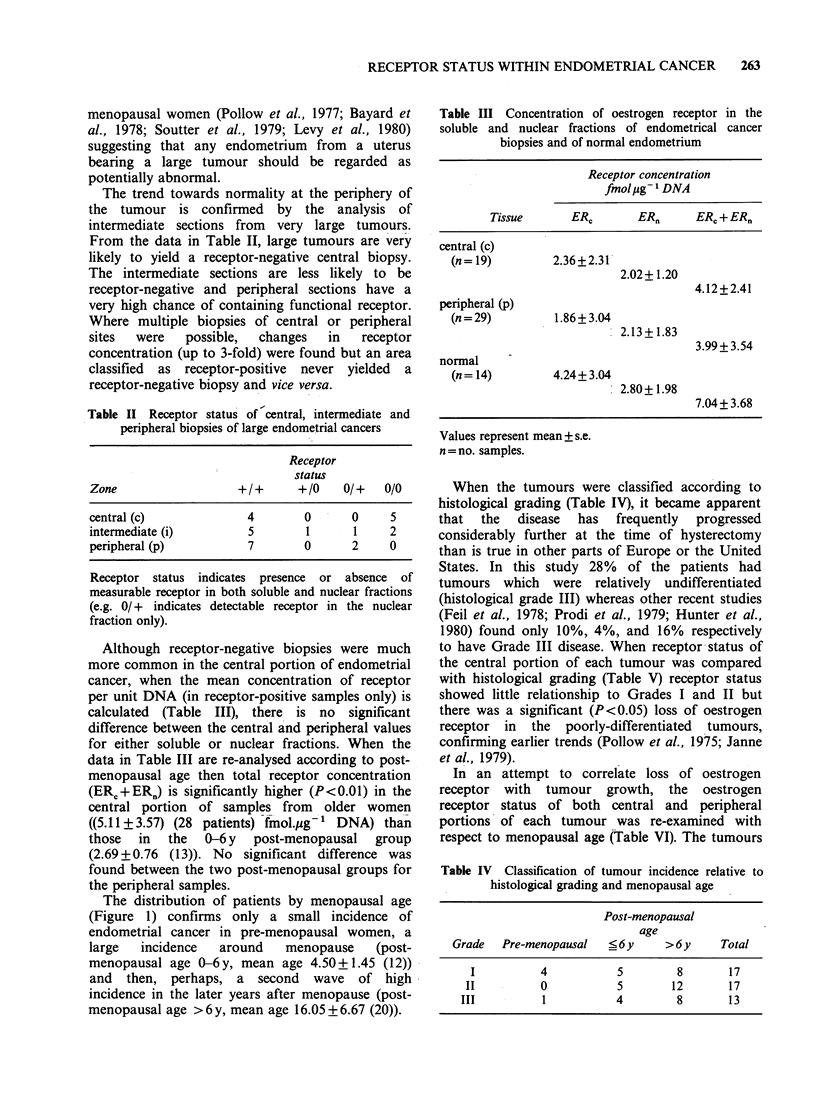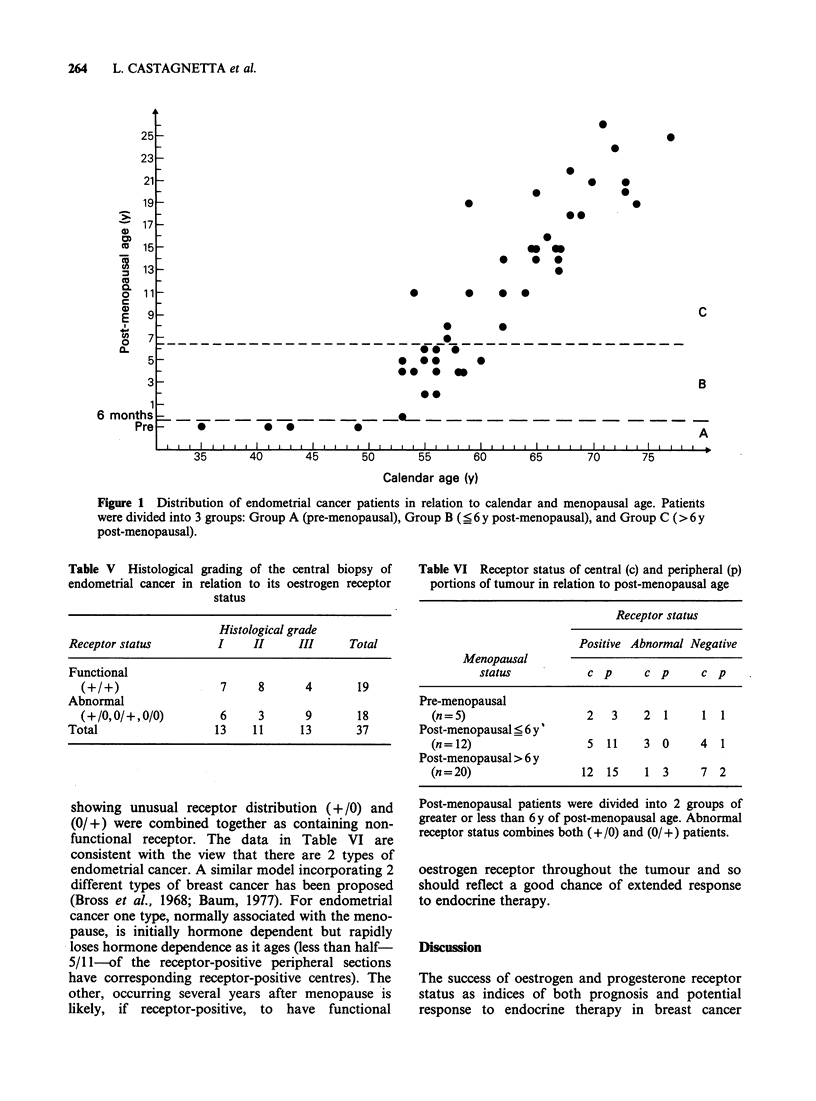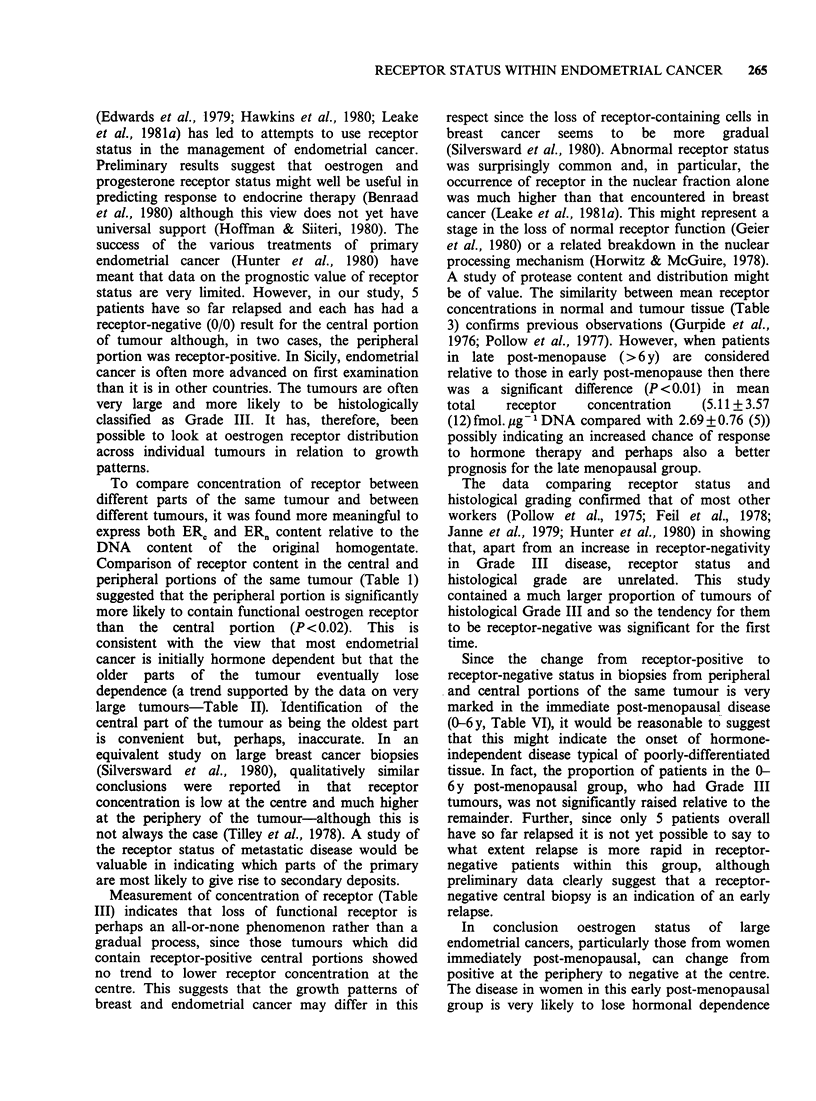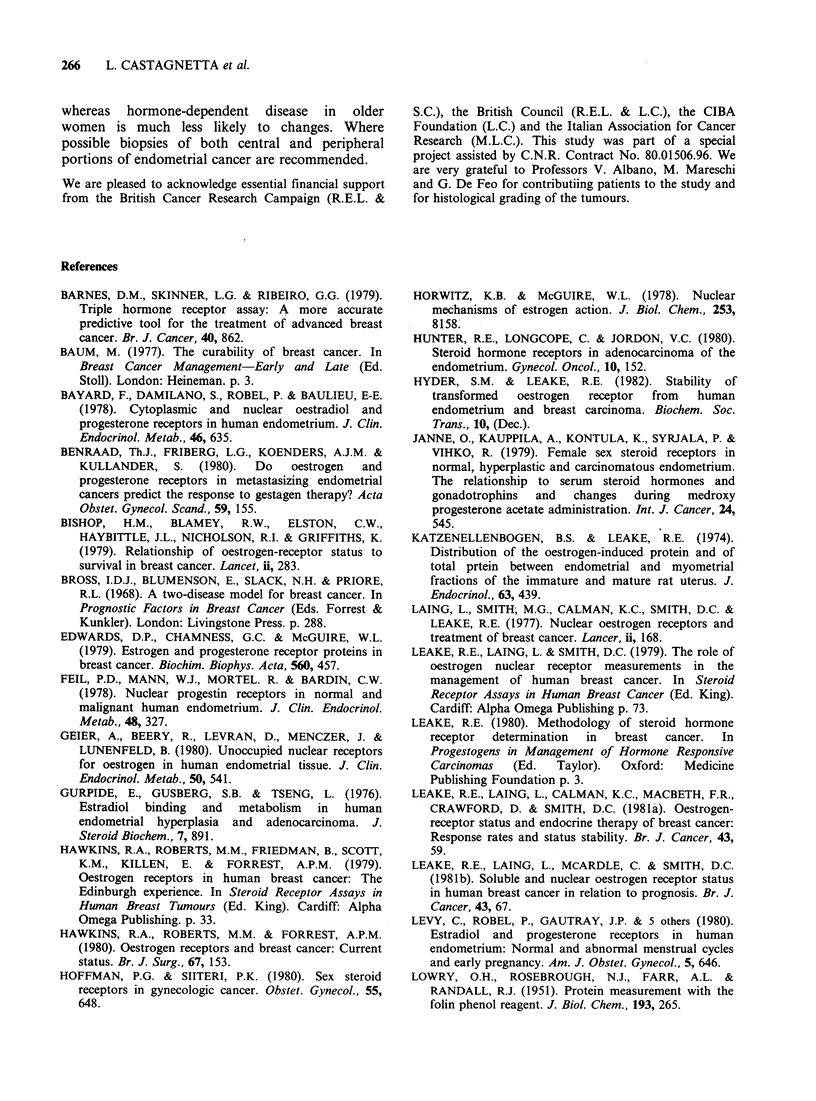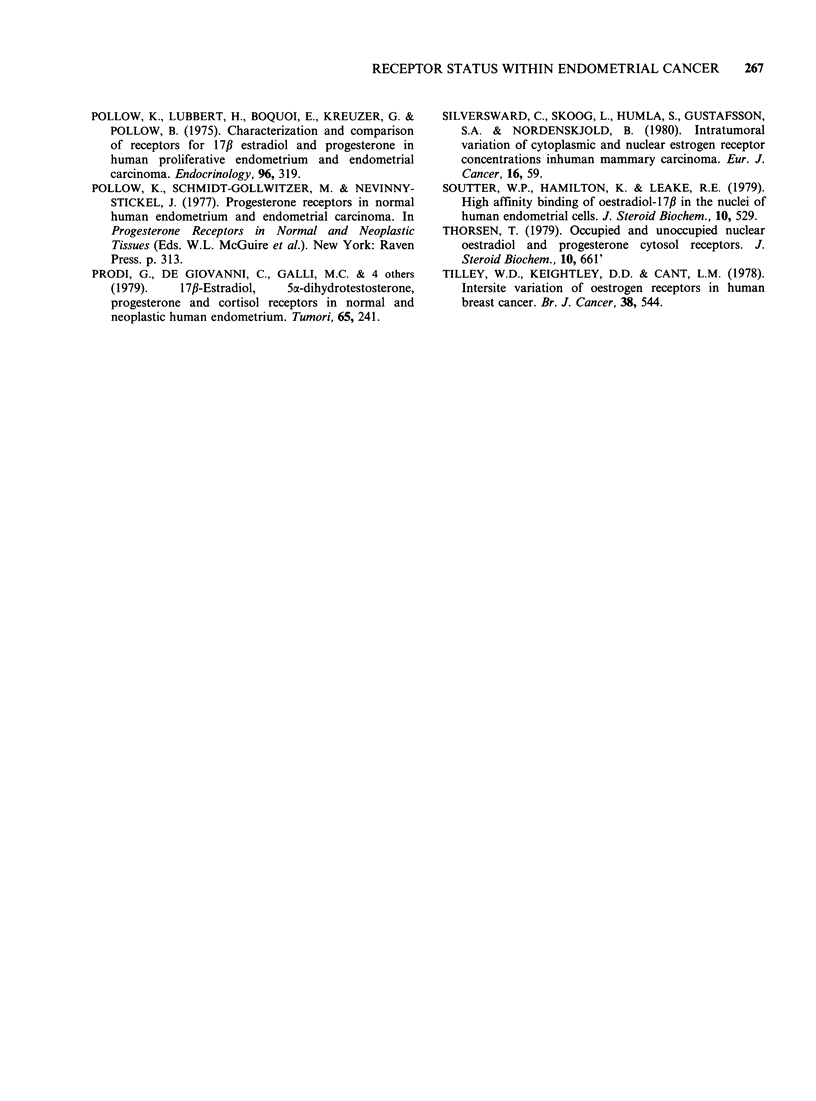# Intra-tumoural variation of oestrogen receptor status in endometrial cancer.

**DOI:** 10.1038/bjc.1983.34

**Published:** 1983-02

**Authors:** L. Castagnetta, M. Lo Casto, T. Mercadante, L. Polito, S. Cowan, R. E. Leake

## Abstract

Soluble and nuclear oestrogen receptor status was determined in both the central and peripheral portions of tumour for 37 cases of adenocarcinoma of the endometrium. Of these, 29 had functional receptor in the peripheral biopsy, but only 19 retained functional receptor in the centre. Six of the 10 patients whose tumours showed this difference came from the group of 12 patients who were immediately post-menopausal (4.50 +/- 1.45 y post-menopausal age). Receptor status was not related to tumour classification into histological grades I and II. However, receptor-negative central biopsies were significantly more likely (P less than 0.05) to be Grade III. Early relapse was also related to a receptor-negative central biopsy.


Br. J. Cancer (1983), 47, 261–267

### Intra-tumoural variation of oestrogen receptor status in endometrial cancer

L. Castagnetta*, M. Lo Casto*, T. Mercadante*, L. Polito*, S. Cowan† & R.E. Leake†

*Istituto di Chimica Biologica and Clinica Ostetrica e Ginecologia II, Facolta di Medicina e Chirurgia—Policlinico, 90127, Italy. †Department of Biochemistry, University of Glasgow, Glasgow, G12 8QQ.

**Summary** Soluble and nuclear oestrogen receptor status was determined in both the central and peripheral portions of tumour for 37 cases of adenocarcinoma of the endometrium. Of these, 29 had functional receptor in the peripheral biopsy, but only 19 retained functional receptor in the centre. Six of the 10 patients whose tumours showed this difference came from the group of 12 patients who were immediately post-menopausal ($4.50 \pm 1.45$ y post-menopausal age). Receptor status was not related to tumour classification into histological grades I and II. However, receptor-negative central biopsies were significantly more likely ($P<0.05$) to be Grade III. Early relapse was also related to a receptor-negative central biopsy.

Several studies have approached the problem of intra-tumoural variation in oestrogen receptor status in breast cancer (Tilley *et al.*, 1978; Silversward *et al.*, 1980). Others have demonstrated the advantages of measuring receptor in both soluble and pellet fractions of each biopsy (Laing *et al.*, 1977; Barnes *et al.*, 1979; Thorsen, 1979). With the increased use of receptor status, both as an index of prognosis (Bishop *et al.*, 1979; Hawkins *et al.*, 1980; Leake *et al.*, 1981*b*), and in selection of therapy (Edwards *et al.*, 1979; Hawkins *et al.*, 1979; Leake *et al.*, 1981*a*), the optimum choice of tumour section for receptor assay has become very important in breast cancer management. Since it has been proposed that steroid receptor status has similar potential in the management of endometrial cancer (Pollow *et al.*, 1975; Bayard *et al.*, 1978; Feil *et al.*, 1978; Prodi *et al.*, 1979), a close study of tumour sampling problems is indicated. We present an analysis of the intra-tumoural variation of both soluble and nuclear oestrogen receptor status in adenocarcinoma of the endometrium.

We report that comparison of the intra- and inter-tumour soluble and nuclear oestrogen receptor status is best made when expressed per unit DNA. Our results indicate that the concentration of receptor in each fraction of receptor-positive tumour is similar to that in normal endometrium. We report that tumours of histological grade III, more frequent in Sicily than elsewhere, are significantly more likely to be receptor-negative although receptor status does not distinguish Grades I and II. A significant proportion of tumours with receptor-positive peripheral biopsies have receptor-negative central biopsies, particularly in a group of patients who are immediately post-menopausal.

### Materials and methods

All patients attended the Obstetric and Gynaecologic Clinic of the Policlinico or the Cancer Hospital Centre, Palermo. All tissue was obtained after hysterectomy. Obviously nectrotic tissue was discarded and then parallel sections removed for pathological examination and receptor assay. The latter were stored in 0.25 M Sucrose, 1.5 mM $MgCl_2$, 10 mM HEPES, pH 7.4, 50% Glycerol (v/v) at $-20°C$ until use. This storage procedure has been found to maintain both concentration (Leake *et al.*, 1979; Leake, 1980) and molecular form (Hyder & Leake, in press) of oestrogen receptor for up to 60 days.

Where possible (37 cases out of 47) tumour tissue was sub-divided according to its location *in situ*. Sub-sections were identified as (1) peripheral (p)—that taken from the so-called "growing" edge of the tumour (care was taken to avoid sections from areas of myometrial infiltration); and (2) central (c)—that taken from non-peripheral and, supposedly, older parts of the tumour. The term "central" is used for convenience only, since a physical centre for endometrial cancer is often difficult to define. Occasional intermediate pieces (i) were also retained from larger tumours in which the central part could be legitimately identified.

Correspondence: R.E. Leake

Received 29 August 1982; accepted 13 October 1982.

0007-0920/83/020261-07 $02.00

© The Macmillan Press Ltd., 1983

Oestrogen receptor assays were carried out on both the soluble and pellet fractions of each section of tissue. The assay (incorporating a 7-point Scatchard plot) measured specific, high affinity binding sites only and has been described in detail (Leake, 1980; Leake *et al.*, 1981*a*). Briefly, [$^3$H]-oestradiol was used as radioligand and specific binding was suppressed in parallel incubations using 100-fold excess of diethylstilboestrol. Unbound steroid was removed with dextran-coated charcoal (soluble fraction) or by extensive washing on Whatman GF/C discs (pellet fraction). Protein content was determined by the method of Lowry *et al.* (1951) and DNA by a modification of the method of Burton (Katzenellenbogen & Leake, 1974). For a biopsy to be classified as positive, receptor content had to exceed 0.2 fmol $\mu g^{-1}$ DNA.

Tumour tissue, confirmed pathologically as adenocarcinoma of the endometrium, was taken from 47 patients of whom 42 were post-menopausal. The post-menopausal group was sub-divided into (1) those greater than and (2) those less than 6 y of post-menopausal age. Where possible, sections of pathologically "normal" endometrium was also dissected out. All other procedures were carried out as previously described (Leake *et al.*, 1981*a*). Where appropriate, data are quoted as mean ±s.e. (no. of samples).

### Results

The concentration of specific oestrogen receptor, as calculated by Scatchard analysis, in the soluble fraction is normally expressed per unit cytosol protein (Bayard *et al.*, 1978; Soutter *et al.*, 1979; Hawkins *et al.*, 1980). However, all results were also calculated relative to the DNA content of the original homogenate. In anticipation of subsequent statistical analysis, the "goodness of fit" to a normal distribution curve was calculated for receptor data relative to both protein and DNA. For soluble receptor ($ER_c$), distribution was abnormal when the data were expressed per unit protein ($P<0.001$) whereas expressed per unit DNA the fit was much better ($0.5<P<0.75$). For nuclear receptor ($ER_n$) and for total receptor ($ER_c+ER_n$) expressed per unit DNA a good fit was observed ($P=0.75$ and 0.90, respectively). For this reason, receptor concentrations for both soluble and nuclear fraction is reported per unit DNA.

Oestrogen receptor status of the 37 cases of endometrial cancer for which it was possible to obtain separate central and peripheral portions is presented in Table I. Table I also shows the receptor status of the samples of pathologically "normal" tissue. $ER_c$ was found in 51% (19/37) of the central biopsies and 78% (29/37) of the peripheral samples. Figures reported by others (70–80% soluble receptor-positive, see Janne *et al.*, 1979; Hunter *et al.*, 1980) suggest that they are only sampling peripheral regions or, probably, small tumours. Functional oestrogen receptor, for the purposes of discussion, has been defined as the presence of measurable receptor in both the soluble and nuclear fractions of a biopsy (Leake, 1980; Leake *et al.*, 1981*a*). It has been demonstrated in breast cancer that the response rate to endocrine therapy is elevated to ~70% by the inclusion of the $ER_n$ assay (Leake *et al.*, 1981*a*) and that occurrence of both $ER_c$ and $ER_n$ usually coincides with that of progesterone receptor (Barnes *et al.*, 1979). Loss of functional oestrogen receptor is seen to be common in the centre of large endometrial tumours (18/37) but is much less common in the peripheral regions (8/37). This difference is significant ($P<0.02$). It is unlikely that this observation could be explained in terms of necrosis at the centre of the tumour since obviously necrotic tissue was excluded during initial sampling and subsequent histological examination of the parallel sections never indicated extensive necrosis in the sections retained. Perhaps, the most likely explanation for the initial loss of detectable receptor from the older parts of the tumour is the cut-back in synthesis of "luxury" proteins in response to the fall in blood supply. If the blood supply is not restored then the cells may become permanently autonomous. The number of patients (437 for central biopsies) having receptor only in the nucleus is high compared to the equivalent data for breast cancer (6%) (Leake *et al.*, 1981*a*) but this represents only a small number of patients. The supposedly normal tissue shows some abnormalities not usually found in curettage tissue from pre-

**Table I** Soluble and nuclear oestrogen receptor status of central and peripheral portions of endometrial carcinoma and of normal endometrium

| *Tissue type* | *Receptor status* $ER_c/ER_n$ +/+ | +/0 | 0/+ | 0/0 |
|---|---|---|---|---|
| central (c) (*n*=37) | 19 | 2 | 4 | 12 |
| peripheral (p) (*n*=37) | 29 | 1 | 3 | 4 |
| normal (*n*=18) | 15 | 0 | 1 | 2 |

Oestrogen receptor status is reported as positive if the Scatchard plot satisfied the usual criteria (Leake *et al.*, 1981*a*) and receptor concentration exceeded 0.2 fM $\mu g^{-1}$ DNA.

menopausal women (Pollow *et al.*, 1977; Bayard *et al.*, 1978; Soutter *et al.*, 1979; Levy *et al.*, 1980) suggesting that any endometrium from a uterus bearing a large tumour should be regarded as potentially abnormal.

The trend towards normality at the periphery of the tumour is confirmed by the analysis of intermediate sections from very large tumours. From the data in Table II, large tumours are very likely to yield a receptor-negative central biopsy. The intermediate sections are less likely to be receptor-negative and peripheral sections have a very high chance of containing functional receptor. Where multiple biopsies of central or peripheral sites were possible, changes in receptor concentration (up to 3-fold) were found but an area classified as receptor-positive never yielded a receptor-negative biopsy and *vice versa*.

**Table II** Receptor status of central, intermediate and peripheral biopsies of large endometrial cancers

| Zone | Receptor status +/+ | +/0 | 0/+ | 0/0 |
|---|---|---|---|---|
| central (c) | 4 | 0 | 0 | 5 |
| intermediate (i) | 5 | 1 | 1 | 2 |
| peripheral (p) | 7 | 0 | 2 | 0 |

Receptor status indicates presence or absence of measurable receptor in both soluble and nuclear fractions (e.g. 0/+ indicates detectable receptor in the nuclear fraction only).

Although receptor-negative biopsies were much more common in the central portion of endometrial cancer, when the mean concentration of receptor per unit DNA (in receptor-positive samples only) is calculated (Table III), there is no significant difference between the central and peripheral values for either soluble or nuclear fractions. When the data in Table III are re-analysed according to post-menopausal age then total receptor concentration ($ER_c+ER_n$) is significantly higher ($P<0.01$) in the central portion of samples from older women (($5.11 \pm 3.57$) (28 patients) fmol.$\mu g^{-1}$ DNA) than those in the 0–6 y post-menopausal group ($2.69 \pm 0.76$ (13)). No significant difference was found between the two post-menopausal groups for the peripheral samples.

The distribution of patients by menopausal age (Figure 1) confirms only a small incidence of endometrial cancer in pre-menopausal women, a large incidence around menopause (post-menopausal age 0–6 y, mean age $4.50 \pm 1.45$ (12)) and then, perhaps, a second wave of high incidence in the later years after menopause (post-menopausal age >6 y, mean age $16.05 \pm 6.67$ (20)).

**Table III** Concentration of oestrogen receptor in the soluble and nuclear fractions of endometrical cancer biopsies and of normal endometrium

| Tissue | Receptor concentration fmol $\mu g^{-1}$ DNA $ER_c$ | $ER_n$ | $ER_c+ER_n$ |
|---|---|---|---|
| central (c) ($n=19$) | $2.36 \pm 2.31$ | $2.02 \pm 1.20$ | $4.12 \pm 2.41$ |
| peripheral (p) ($n=29$) | $1.86 \pm 3.04$ | $2.13 \pm 1.83$ | $3.99 \pm 3.54$ |
| normal ($n=14$) | $4.24 \pm 3.04$ | $2.80 \pm 1.98$ | $7.04 \pm 3.68$ |

Values represent mean ± s.e.
$n$ = no. samples.

When the tumours were classified according to histological grading (Table IV), it became apparent that the disease has frequently progressed considerably further at the time of hysterectomy than is true in other parts of Europe or the United States. In this study 28% of the patients had tumours which were relatively undifferentiated (histological grade III) whereas other recent studies (Feil *et al.*, 1978; Prodi *et al.*, 1979; Hunter *et al.*, 1980) found only 10%, 4%, and 16% respectively to have Grade III disease. When receptor status of the central portion of each tumour was compared with histological grading (Table V) receptor status showed little relationship to Grades I and II but there was a significant ($P<0.05$) loss of oestrogen receptor in the poorly-differentiated tumours, confirming earlier trends (Pollow *et al.*, 1975; Janne *et al.*, 1979).

In an attempt to correlate loss of oestrogen receptor with tumour growth, the oestrogen receptor status of both central and peripheral portions of each tumour was re-examined with respect to menopausal age (Table VI). The tumours

**Table IV** Classification of tumour incidence relative to histological grading and menopausal age

| Grade | Pre-menopausal | Post-menopausal age ≦6 y | >6 y | Total |
|---|---|---|---|---|
| I | 4 | 5 | 8 | 17 |
| II | 0 | 5 | 12 | 17 |
| III | 1 | 4 | 8 | 13 |

**Figure 1** Distribution of endometrial cancer patients in relation to calendar and menopausal age. Patients were divided into 3 groups: Group A (pre-menopausal), Group B ($\leq$6 y post-menopausal), and Group C (>6 y post-menopausal).

**Table V** Histological grading of the central biopsy of endometrial cancer in relation to its oestrogen receptor status

| Receptor status | Histological grade I | II | III | Total |
|---|---|---|---|---|
| Functional (+/+) | 7 | 8 | 4 | 19 |
| Abnormal (+/0, 0/+, 0/0) | 6 | 3 | 9 | 18 |
| Total | 13 | 11 | 13 | 37 |

**Table VI** Receptor status of central (c) and peripheral (p) portions of tumour in relation to post-menopausal age

| Menopausal status | Receptor status: Positive c | Positive p | Abnormal c | Abnormal p | Negative c | Negative p |
|---|---|---|---|---|---|---|
| Pre-menopausal ($n$=5) | 2 | 3 | 2 | 1 | 1 | 1 |
| Post-menopausal $\leq$6 y ($n$=12) | 5 | 11 | 3 | 0 | 4 | 1 |
| Post-menopausal >6 y ($n$=20) | 12 | 15 | 1 | 3 | 7 | 2 |

Post-menopausal patients were divided into 2 groups of greater or less than 6 y of post-menopausal age. Abnormal receptor status combines both (+/0) and (0/+) patients.

showing unusual receptor distribution (+/0) and (0/+) were combined together as containing non-functional receptor. The data in Table VI are consistent with the view that there are 2 types of endometrial cancer. A similar model incorporating 2 different types of breast cancer has been proposed (Bross *et al.*, 1968; Baum, 1977). For endometrial cancer one type, normally associated with the menopause, is initially hormone dependent but rapidly loses hormone dependence as it ages (less than half—5/11—of the receptor-positive peripheral sections have corresponding receptor-positive centres). The other, occurring several years after menopause is likely, if receptor-positive, to have functional oestrogen receptor throughout the tumour and so should reflect a good chance of extended response to endocrine therapy.

### Discussion

The success of oestrogen and progesterone receptor status as indices of both prognosis and potential response to endocrine therapy in breast cancer

(Edwards *et al.*, 1979; Hawkins *et al.*, 1980; Leake *et al.*, 1981*a*) has led to attempts to use receptor status in the management of endometrial cancer. Preliminary results suggest that oestrogen and progesterone receptor status might well be useful in predicting response to endocrine therapy (Benraad *et al.*, 1980) although this view does not yet have universal support (Hoffman & Siiteri, 1980). The success of the various treatments of primary endometrial cancer (Hunter *et al.*, 1980) have meant that data on the prognostic value of receptor status are very limited. However, in our study, 5 patients have so far relapsed and each has had a receptor-negative (0/0) result for the central portion of tumour although, in two cases, the peripheral portion was receptor-positive. In Sicily, endometrial cancer is often more advanced on first examination than it is in other countries. The tumours are often very large and more likely to be histologically classified as Grade III. It has, therefore, been possible to look at oestrogen receptor distribution across individual tumours in relation to growth patterns.

To compare concentration of receptor between different parts of the same tumour and between different tumours, it was found more meaningful to express both $ER_c$ and $ER_n$ content relative to the DNA content of the original homogentate. Comparison of receptor content in the central and peripheral portions of the same tumour (Table 1) suggested that the peripheral portion is significantly more likely to contain functional oestrogen receptor than the central portion ($P<0.02$). This is consistent with the view that most endometrial cancer is initially hormone dependent but that the older parts of the tumour eventually lose dependence (a trend supported by the data on very large tumours—Table II). Identification of the central part of the tumour as being the oldest part is convenient but, perhaps, inaccurate. In an equivalent study on large breast cancer biopsies (Silversward *et al.*, 1980), qualitatively similar conclusions were reported in that receptor concentration is low at the centre and much higher at the periphery of the tumour—although this is not always the case (Tilley *et al.*, 1978). A study of the receptor status of metastatic disease would be valuable in indicating which parts of the primary are most likely to give rise to secondary deposits.

Measurement of concentration of receptor (Table III) indicates that loss of functional receptor is perhaps an all-or-none phenomenon rather than a gradual process, since those tumours which did contain receptor-positive central portions showed no trend to lower receptor concentration at the centre. This suggests that the growth patterns of breast and endometrial cancer may differ in this respect since the loss of receptor-containing cells in breast cancer seems to be more gradual (Silversward *et al.*, 1980). Abnormal receptor status was surprisingly common and, in particular, the occurrence of receptor in the nuclear fraction alone was much higher than that encountered in breast cancer (Leake *et al.*, 1981*a*). This might represent a stage in the loss of normal receptor function (Geier *et al.*, 1980) or a related breakdown in the nuclear processing mechanism (Horwitz & McGuire, 1978). A study of protease content and distribution might be of value. The similarity between mean receptor concentrations in normal and tumour tissue (Table 3) confirms previous observations (Gurpide *et al.*, 1976; Pollow *et al.*, 1977). However, when patients in late post-menopause ($>6$ y) are considered relative to those in early post-menopause then there was a significant difference ($P<0.01$) in mean total receptor concentration ($5.11 \pm 3.57$ (12) fmol. $\mu g^{-1}$ DNA compared with $2.69 \pm 0.76$ (5)) possibly indicating an increased chance of response to hormone therapy and perhaps also a better prognosis for the late menopausal group.

The data comparing receptor status and histological grading confirmed that of most other workers (Pollow *et al.*, 1975; Feil *et al.*, 1978; Janne *et al.*, 1979; Hunter *et al.*, 1980) in showing that, apart from an increase in receptor-negativity in Grade III disease, receptor status and histological grade are unrelated. This study contained a much larger proportion of tumours of histological Grade III and so the tendency for them to be receptor-negative was significant for the first time.

Since the change from receptor-positive to receptor-negative status in biopsies from peripheral and central portions of the same tumour is very marked in the immediate post-menopausal disease (0–6 y, Table VI), it would be reasonable to suggest that this might indicate the onset of hormone-independent disease typical of poorly-differentiated tissue. In fact, the proportion of patients in the 0–6 y post-menopausal group, who had Grade III tumours, was not significantly raised relative to the remainder. Further, since only 5 patients overall have so far relapsed it is not yet possible to say to what extent relapse is more rapid in receptor-negative patients within this group, although preliminary data clearly suggest that a receptor-negative central biopsy is an indication of an early relapse.

In conclusion oestrogen status of large endometrial cancers, particularly those from women immediately post-menopausal, can change from positive at the periphery to negative at the centre. The disease in women in this early post-menopausal group is very likely to lose hormonal dependence whereas hormone-dependent disease in older women is much less likely to changes. Where possible biopsies of both central and peripheral portions of endometrial cancer are recommended.

We are pleased to acknowledge essential financial support from the British Cancer Research Campaign (R.E.L. & S.C.), the British Council (R.E.L. & L.C.), the CIBA Foundation (L.C.) and the Italian Association for Cancer Research (M.L.C.). This study was part of a special project assisted by C.N.R. Contract No. 80.01506.96. We are very grateful to Professors V. Albano, M. Mareschi and G. De Feo for contributiing patients to the study and for histological grading of the tumours.

### References

BARNES, D.M., SKINNER, L.G. & RIBEIRO, G.G. (1979). Triple hormone receptor assay: A more accurate predictive tool for the treatment of advanced breast cancer. *Br. J. Cancer*, **40**, 862.

BAUM, M. (1977). The curability of breast cancer. In *Breast Cancer Management—Early and Late* (Ed. Stoll). London: Heineman. p. 3.

BAYARD, F., DAMILANO, S., ROBEL, P. & BAULIEU, E-E. (1978). Cytoplasmic and nuclear oestradiol and progesterone receptors in human endometrium. *J. Clin. Endocrinol. Metab.*, **46**, 635.

BENRAAD, Th.J., FRIBERG, L.G., KOENDERS, A.J.M. & KULLANDER, S. (1980). Do oestrogen and progesterone receptors in metastasizing endometrial cancers predict the response to gestagen therapy? *Acta Obstet. Gynecol. Scand.*, **59**, 155.

BISHOP, H.M., BLAMEY, R.W., ELSTON, C.W., HAYBITTLE, J.L., NICHOLSON, R.I. & GRIFFITHS, K. (1979). Relationship of oestrogen-receptor status to survival in breast cancer. *Lancet*, **ii**, 283.

BROSS, I.D.J., BLUMENSON, E., SLACK, N.H. & PRIORE, R.L. (1968). A two-disease model for breast cancer. In *Prognostic Factors in Breast Cancer* (Eds. Forrest & Kunkler). London: Livingstone Press. p. 288.

EDWARDS, D.P., CHAMNESS, G.C. & McGUIRE, W.L. (1979). Estrogen and progesterone receptor proteins in breast cancer. *Biochim. Biophys. Acta*, **560**, 457.

FEIL, P.D., MANN, W.J., MORTEL. R. & BARDIN, C.W. (1978). Nuclear progestin receptors in normal and malignant human endometrium. *J. Clin. Endocrinol. Metab.*, **48**, 327.

GEIER, A., BEERY, R., LEVRAN, D., MENCZER, J. & LUNENFELD, B. (1980). Unoccupied nuclear receptors for oestrogen in human endometrial tissue. *J. Clin. Endocrinol. Metab.*, **50**, 541.

GURPIDE, E., GUSBERG, S.B. & TSENG, L. (1976). Estradiol binding and metabolism in human endometrial hyperplasia and adenocarcinoma. *J. Steroid Biochem.*, **7**, 891.

HAWKINS, R.A., ROBERTS, M.M., FRIEDMAN, B., SCOTT, K.M., KILLEN, E. & FORREST, A.P.M. (1979). Oestrogen receptors in human breast cancer: The Edinburgh experience. In *Steroid Receptor Assays in Human Breast Tumours* (Ed. King). Cardiff: Alpha Omega Publishing. p. 33.

HAWKINS, R.A., ROBERTS, M.M. & FORREST, A.P.M. (1980). Oestrogen receptors and breast cancer: Current status. *Br. J. Surg.*, **67**, 153.

HOFFMAN, P.G. & SIITERI, P.K. (1980). Sex steroid receptors in gynecologic cancer. *Obstet. Gynecol.*, **55**, 648.

HORWITZ, K.B. & McGUIRE, W.L. (1978). Nuclear mechanisms of estrogen action. *J. Biol. Chem.*, **253**, 8158.

HUNTER, R.E., LONGCOPE, C. & JORDON, V.C. (1980). Steroid hormone receptors in adenocarcinoma of the endometrium. *Gynecol. Oncol.*, **10**, 152.

HYDER, S.M. & LEAKE, R.E. (1982). Stability of transformed oestrogen receptor from human endometrium and breast carcinoma. *Biochem. Soc. Trans.*, **10**, (Dec.).

JANNE, O., KAUPPILA, A., KONTULA, K., SYRJALA, P. & VIHKO, R. (1979). Female sex steroid receptors in normal, hyperplastic and carcinomatous endometrium. The relationship to serum steroid hormones and gonadotrophins and changes during medroxy progesterone acetate administration. *Int. J. Cancer*, **24**, 545.

KATZENELLENBOGEN, B.S. & LEAKE, R.E. (1974). Distribution of the oestrogen-induced protein and of total prtein between endometrial and myometrial fractions of the immature and mature rat uterus. *J. Endocrinol.*, **63**, 439.

LAING, L., SMITH, M.G., CALMAN, K.C., SMITH, D.C. & LEAKE, R.E. (1977). Nuclear oestrogen receptors and treatment of breast cancer. *Lancer*, **ii**, 168.

LEAKE, R.E., LAING, L. & SMITH, D.C. (1979). The role of oestrogen nuclear receptor measurements in the management of human breast cancer. In *Steroid Receptor Assays in Human Breast Cancer* (Ed. King). Cardiff: Alpha Omega Publishing p. 73.

LEAKE, R.E. (1980). Methodology of steroid hormone receptor determination in breast cancer. In *Progestogens in Management of Hormone Responsive Carcinomas* (Ed. Taylor). Oxford: Medicine Publishing Foundation p. 3.

LEAKE, R.E., LAING, L., CALMAN, K.C., MACBETH, F.R., CRAWFORD, D. & SMITH, D.C. (1981a). Oestrogen-receptor status and endocrine therapy of breast cancer: Response rates and status stability. *Br. J. Cancer*, **43**, 59.

LEAKE, R.E., LAING, L., MCARDLE, C. & SMITH, D.C. (1981b). Soluble and nuclear oestrogen receptor status in human breast cancer in relation to prognosis. *Br. J. Cancer*, **43**, 67.

LEVY, C., ROBEL, P., GAUTRAY, J.P. & 5 others (1980). Estradiol and progesterone receptors in human endometrium: Normal and abnormal menstrual cycles and early pregnancy. *Am. J. Obstet. Gynecol.*, **5**, 646.

LOWRY, O.H., ROSEBROUGH, N.J., FARR, A.L. & RANDALL, R.J. (1951). Protein measurement with the folin phenol reagent. *J. Biol. Chem.*, **193**, 265.

POLLOW, K., LUBBERT, H., BOQUOI, E., KREUZER, G. & POLLOW, B. (1975). Characterization and comparison of receptors for 17$\beta$ estradiol and progesterone in human proliferative endometrium and endometrial carcinoma. *Endocrinology*, **96**, 319.

POLLOW, K., SCHMIDT-GOLLWITZER, M. & NEVINNY-STICKEL, J. (1977). Progesterone receptors in normal human endometrium and endometrial carcinoma. In *Progesterone Receptors in Normal and Neoplastic Tissues* (Eds. W.L. McGuire *et al.*). New York: Raven Press. p. 313.

PRODI, G., DE GIOVANNI, C., GALLI, M.C. & 4 others (1979). 17$\beta$-Estradiol, 5$\alpha$-dihydrotestosterone, progesterone and cortisol receptors in normal and neoplastic human endometrium. *Tumori*, **65**, 241.

SILVERSWARD, C., SKOOG, L., HUMLA, S., GUSTAFSSON, S.A. & NORDENSKJOLD, B. (1980). Intratumoral variation of cytoplasmic and nuclear estrogen receptor concentrations inhuman mammary carcinoma. *Eur. J. Cancer*, **16**, 59.

SOUTTER, W.P., HAMILTON, K. & LEAKE, R.E. (1979). High affinity binding of oestradiol-17$\beta$ in the nuclei of human endometrial cells. *J. Steroid Biochem.*, **10**, 529.

THORSEN, T. (1979). Occupied and unoccupied nuclear oestradiol and progesterone cytosol receptors. *J. Steroid Biochem.*, **10**, 661'

TILLEY, W.D., KEIGHTLEY, D.D. & CANT, L.M. (1978). Intersite variation of oestrogen receptors in human breast cancer. *Br. J. Cancer*, **38**, 544.